# Lactate: Fueling the fire starter

**DOI:** 10.1002/wsbm.1474

**Published:** 2019-12-16

**Authors:** Michelangelo Certo, Giancarlo Marone, Amato de Paulis, Claudio Mauro, Valentina Pucino

**Affiliations:** ^1^ Institute of Inflammation and Ageing, College of Medical and Dental Sciences University of Birmingham Birmingham UK; ^2^ Department of Public Health University of Naples Federico II Naples Italy; ^3^ Ospedale dei Colli, Hospital Pharmacy Naples Italy; ^4^ Department of Translational Medical Sciences (DISMET) University of Naples Federico II Naples Italy; ^5^ Center for Basic and Clinical Immunology Research (CISI), School of Medicine University of Naples Federico II Naples Italy; ^6^ Institute of Cardiovascular Sciences, College of Medical and Dental Sciences University of Birmingham Birmingham UK; ^7^ Institute of Metabolism and Systems Research, College of Medical and Dental Sciences University of Birmingham Birmingham UK; ^8^ Rheumatology Research Group, Institute for Inflammation and Ageing, College of Medical and Dental Sciences Queen Elizabeth Hospital, University of Birmingham Birmingham UK

**Keywords:** immunity, inflammation, lactate, metabolism, tumor

## Abstract

It is becoming increasingly appreciated that intermediates of metabolic pathways, besides their anabolic and catabolic functions, can act as signaling molecules and influence the outcome of immune responses. Although lactate was previously considered as a waste product of glucose metabolism, accumulating evidence has highlighted its pivotal role in regulating diverse biological processes, including immune cell polarization, differentiation and effector functions. In addition, lactate is a key player in modulating tumor immune surveillance. Hence, targeting lactate‐induced signaling pathways is a promising tool to reduce inflammation, to prevent autoimmunity and to restore anti‐tumor immune response.

This article is characterized under:

Biological Mechanisms > Metabolism

## Graphical abstract

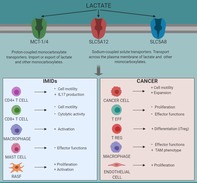

## 1 INTRODUCTION

**Figure 1:**
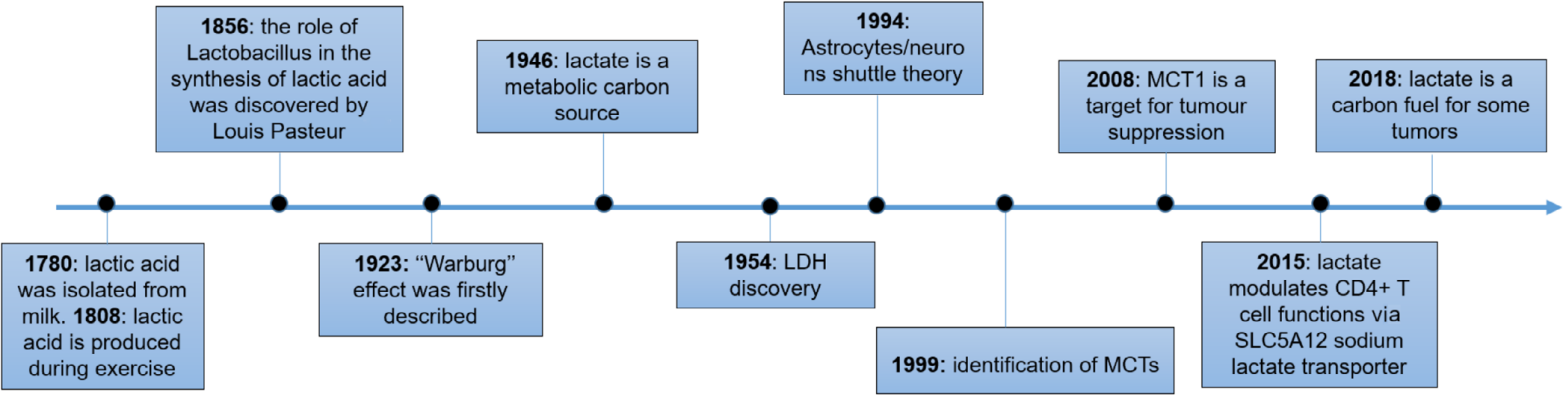
The history of lactate. Lactic acid was isolated for the first time by the chemist Carl Wilhelm Scheele in 1780 from sour milk. Indeed, the name lactate originates from the Latin word lac, which means milk. In 1808, Jöns Jacob Berzelius discovered that lactic acid (l‐lactate) is produced in muscles during exercise. In 1856, the role of *Lactobacillus* in the synthesis of lactate was discovered by Pasteur (1861) and only 20 years later in 1873 lactate molecular structure was resolved by Johannes Wislicenus (Nalbandian & Takeda, 2016). In 1923, Otto Warburg observed that cancer cells were characterized by accelerated glycolysis and excessive lactate generation even under fully oxygenated conditions (Warburg, 1956). His discovery was subsequently named the “Warburg Effect” by Efraim Racker in 1972. In 1946 lactate was identified as a major gluconeogenic precursor acting as shuttle in both muscle and liver (Cori & Cori, 1946). Later in 1954, LDH, the enzyme responsible for lactate production was found elevated in cancer (Hill & Levi, 1954) and in 1994 lactate was identified as major carbon source in the brain facilitating the interconnections between astrocytes and neurons (Pellerin & Magistretti, 1994). MCTs were firstly described as lactate transporters (Halestrap & Price, 1999) and MCT1 was later identified as target for tumor suppression (Sonveaux et al., 2008). Recent studies have emerged describing lactate as immune modulator in inflammatory disorders and as key carbon fuel for some tumors (Colegio et al., 2014; Faubert et al., 2017; Haas et al., 2015; Hui et al., 2017; Pucino et al., 2019)

Lactate, produced at the end of glycolysis, is a metabolite described for the first time at the beginning of the 20th century (Corbet & Feron, 2017; Figure 1). Its physiological concentration is in the range of 1.5–3 mM in blood and tissues. However, lactate levels can increase to 10 mM in several inflammatory conditions and to a concentration of 30–40 mM in tumors (Colegio et al., 2014; Haas et al., 2015; Hirschhaeuser, Sattler, & Mueller‐Klieser, 2011). Elevated amounts of lactate are produced as a consequence of increased glycolysis in proliferating cells (Warburg effect) via lactate dehydrogenase (LDH), an enzyme that also regenerates NAD^+^ from the reduced form of nicotinamide adenine dinucleotide (NADH), allowing a steady flow of glycolysis (Bonuccelli et al., 2010; Warburg, 1956). Lactate is present in solution either as lactic acid at low pH or as a salt (i.e., sodium lactate) at higher pH, with a pKa of 3.83 (Haas et al., 2015). During hypoxia (i.e., inflammation and cancer) lactate accumulates as a result of increased cell turnover and glycolytic engagement subsequent to the increased hypoxia‐inducible factor (HIF)‐1α response to low oxygen tension (Corcoran & O'Neill, 2016; Lee et al., 2015; Semenza, Roth, Fang, & Wang, 1994). Hypoxic conditions result in prolyl hydroxylase domain (PHD) inhibition and an attenuation of HIF‐1α hydroxylation and proteasomal degradation. HIF‐1α accumulates, translocates to the nucleus, and increases transcription of glycolytic genes. HIF‐1α activates pyruvate dehydrogenase kinase‐1 which phosphorylates and inactivates pyruvate dehydrogenase (PDH) (Kim, Tchernyshyov, Semenza, & Dang, 2006). PDH is normally responsible for the oxidation of pyruvate to acetyl‐CoA for mitochondrial oxidation and its inactivation limits the oxidative disposal of pyruvate favoring the diversion of the glycolytic flux to lactate. Inactivation of PDH by HIF‐1α is well known in exercise metabolism characterized by high glycolytic flux and lactate production (Brooks, 1986, 2009). During high‐intensity intermittent physical exercise, muscle can cope with glycolytic stress by shuttling lactate among tissues and using it as carbon source for energy production. For instance, lactate is a major gluconeogenic precursor acting as a shuttle in both muscle and liver (Cori & Cori, 1946). Indeed, lactate flux is metabolically beneficial through supplying carbons to gluconeogenesis during exercise (Brooks, 2009, 2018). In cancer and chronic inflammation, the persistent stimulation of glycolysis for the necessity of carbon sources is maladaptive and leads to an unbalanced shuttle mechanism between the tissues (Brooks, 2018). Lactate has also been shown to be important for normal brain physiology, through the astrocyte‐neuron lactate shuttle. In this model, described in 1994, the neurotransmitter glutamate released in the synapse, triggers glucose uptake and therefore lactate production by astrocytes; lactate so produced is then utilized by neurons as a source of energy (Magistretti & Allaman, 2018; Pellerin & Magistretti, 1994).

## 2 LACTATE TRANSPORT

**Figure 2:**
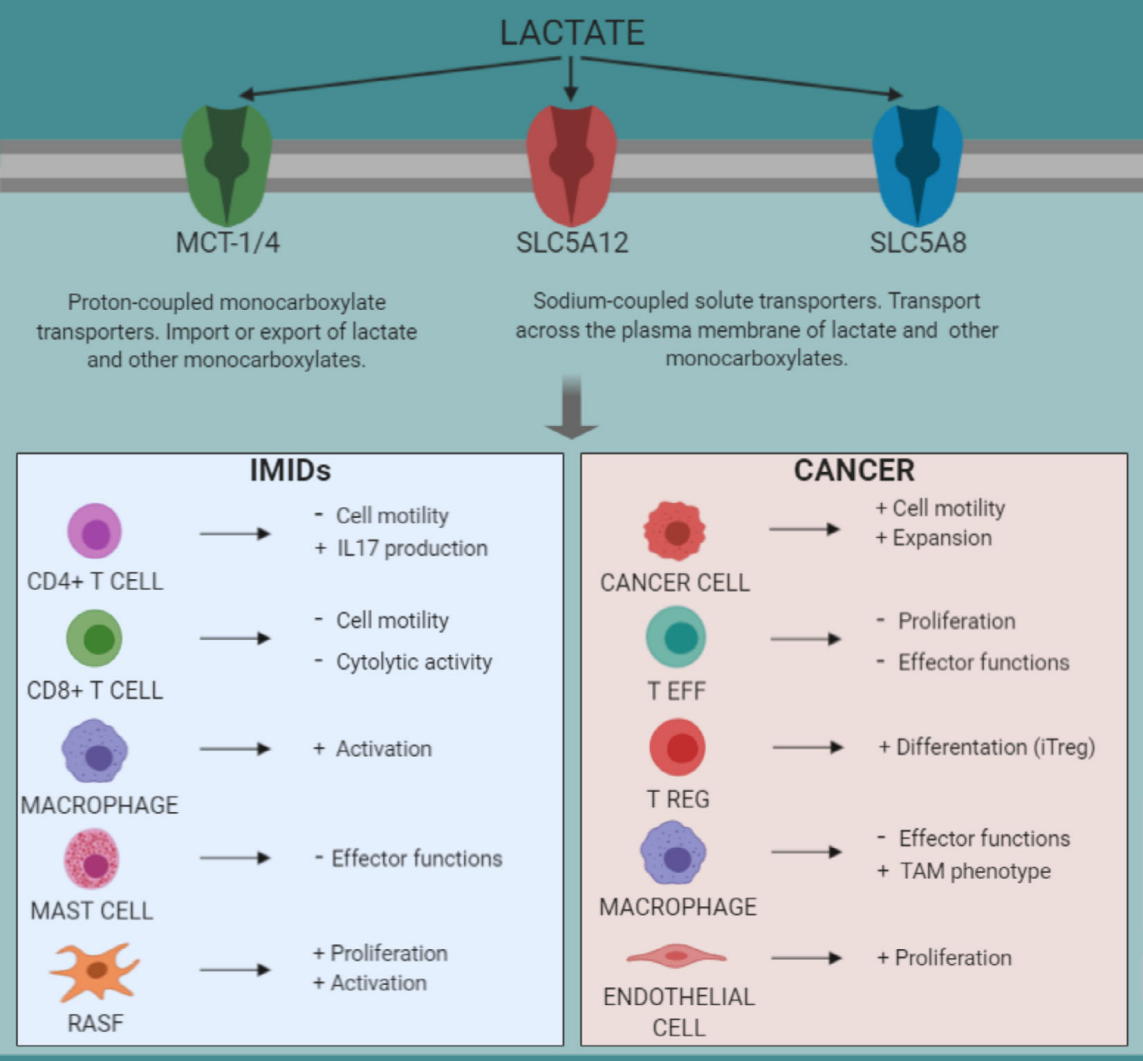
Lactate modulates immune cell functions in immune‐mediated inflammatory disorders and cancer. Immune cells “sense” high concentration of lactate which accumulates at the site of inflammation or tumor as result of accelerated metabolism of immune, stromal, or cancer cells. Lactate is taken up through specific transporters expressed on the cell membrane and modulates immune responses, including activation, differentiation, proliferation, migration, and cytokine production. These events promote the establishment of a chronic inflammatory process in IMIDs and induce tumor growth and metastatic spread in cancer

Lactate uptake and release requires the presence of transporters on the cell plasma membrane. Four members of the solute carrier 16a family of 12‐membrane pass, proton‐linked monocarboxylic acid symporters (i.e., monocarboxylic transporter 1 [MCT1, SLC16A1], MCT2 [SLC16A7], MCT3 [SLC16A8], and MCT4 [SLC16A3]), and two sodium‐coupled lactate cotransporters (SLC5A12, SLC5A8) have been studied in more detail (Table 1, Figure 2; Halestrap & Price, 1999; Pucino, Cucchi, & Mauro, 2018; Srinivas et al., 2005). These channels share conserved sequence motifs but display different affinity for lactate and other monocarboxylates (Doherty & Cleveland, 2013; Hirschhaeuser et al., 2011). The transport depends on pH, intra‐ and extracellular lactate concentration as well as other substrates such as pyruvate, butyrate, etc. (Doherty & Cleveland, 2013; Hirschhaeuser et al., 2011).

**Table 1:** Lactate transporters

Transporter	Function	High affinity substrates	Cell expression
SLC16A1/MCT1	H^+^‐coupled electroneutral transporter	Lactate, pyruvate, ketone bodies	Epithelial cells, macrophages, CD8+ lymphocytes, cancer cells
SLC16A7/MCT2	H^+^‐coupled electroneutral transporter	Pyruvate, lactate, ketone bodies	Epithelial cells, cancer cells
SLC16A8/MCT3	H^+^‐coupled electroneutral transporter	Lactate	Epithelial cells (highly expressed in the retina)
SLC16A3/MCT4	H^+^‐coupled electroneutral transporter	Lactate, pyruvate, ketone bodies	Epithelial cells, fibroblasts, macrophages, cancer cells
SLC5A8/SMCT1	Na^+^‐coupled electroneutral transporter	Lactate, pyruvate, propionate, butyrate, nicotinate, and short‐chain fatty acids	Epithelial cells (mainly kidney, intestine, brain)
SLC5A12/SMCT2	Na^+^‐coupled electroneutral transporter	Lactate, pyruvate, propionate, butyrate, nicotinate, and short‐chain fatty acids	Epithelial cells (mainly kidney, intestine, brain), CD4+ lymphocytes

MCTs have been identified in all eukaryotic organisms and catalyze the proton‐linked transport of a wide variety of substrates (Perez‐Escuredo, Dadhich, et al., 2016) such as pyruvate, lactate and ketone bodies (acetoacetate and d‐β‐hydroxybutyrate), across the plasma membrane (Halestrap & Meredith, 2004; Halestrap & Price, 1999). Other identified MCTs are MCT5‐9, which show high affinity for other substrates such as thyroid hormones, ketone bodies, bumetanide and small aminoacids (Halestrap & Meredith, 2004; Hugo et al., 2012; Murakami et al., 2005; Suhre et al., 2011; Visser, van Mullem, Jansen, & Visser, 2011). The substrates and functions of the other MCT family members are still under investigation.

MCTs are expressed in a wide range of tissues (such as brain, skeletal muscle, heart, bowel, and liver) and display many physiological functions. In particular, they play a pivotal role in the control of glucose metabolism, and in the regulation of many biological functions including spermatogenesis, pancreatic β cell activity, thyroid hormone metabolism and drug transport (Perez‐Escuredo, Van Hee, et al., 2016).

MCTs are important regulators of intracellular lactate and pH; in particular lactate is one of the main substrates of MCT1–4. Indeed, highly glycolytic cells (i.e., during inflammation or tumors) utilize MCT transporters to export or import lactate (Bonen, 2001; Halestrap & Meredith, 2004; Huang et al., 2017; Sonveaux et al., 2008; Van Hee, Perez‐Escuredo, Cacace, Copetti, & Sonveaux, 2015).

All MCTs are capable of facilitating either import or export of lactate with substrate gradient being the main factor driving directionality. However, several studies have identified MCT4 to be responsible for its export in to the extracellular space while MCT1 regulates lactate import across the plasma membrane in the majority of the tissues where lactate is produced. Here, lactate can be used as a substrate to fuel other metabolic pathways (Faubert et al., 2017; Halestrap, 2012; Halestrap & Meredith, 2004; Perez‐Escuredo, Dadhich, et al., 2016).

In many cancer cells with an oxidative metabolic fingerprint, MCT1 is the most expressed MCT isoform (Kennedy & Dewhirst, 2010; Sonveaux et al., 2008). However, in glycolytic cancer cells or white muscle fibers and astrocytes, MCT4 is expressed at higher level than MCT1 (Baltazar et al., 2014; Sonveaux et al., 2008; Ullah, Davies, & Halestrap, 2006). This different MCTs expression allows the shuttling of this metabolite between cells and tissues with different metabolic behaviors. Such phenomenon has been described in the skeletal muscle where glycolytic/white fibers export lactate through MCT4 and oxidative/red fibers import lactate through MCT1 to fuel the tricarboxylic acid cycle (TCA) (Juel & Halestrap, 1999). A similar mechanism has been reported in cancer where lactate is exchanged between glycolytic/hypoxic and oxidative/oxygenated malignant cells (Sonveaux et al., 2008). In the brain, glycolytic oligodendrocytes and astrocytes export lactate through MCT1 and MCT4 to fuel oxidative neurons expressing MCT2 (Brooks, 2009; Funfschilling et al., 2012; Pellerin & Magistretti, 2012; Saab, Tzvetanova, & Nave, 2013). MCT2 and MCT4 show a high intracellular expression suggesting a possible role in mediating monocarboxylate transport across the membranes of intracellular vesicles or organelles (Afonso et al., 2015; Baltazar et al., 2014). In contrast to MCTs, which function as H^+^‐coupled electroneutral transporters, sodium‐coupled monocarboxylate transporters (SMCTs) function as Na^+^‐coupled transporters with a substrate ratio Na^+^/monocarboxylate ≥2. Two members of the SMCT family have been identified, the high‐affinity transporter SMCT1 (SLC5A8) and the low‐affinity SMCT2 (SLC5A12) (Rodriguez et al., 2002; Srinivas et al., 2005). The SLC5A8 gene was originally identified in the kidney (Rodriguez et al., 2002) and subsequently detected in the intestine, salivary gland, thyroid gland, brain, and retina (Ganapathy et al., 2008). In these tissues, SLC5A8 mediates the transport of monocarboxylic acids such as lactate, pyruvate, propionate, butyrate, nicotinate, and short‐chain fatty acids similar to MCTs with a high affinity (Gopal et al., 2005; Morris & Felmlee, 2008).

SLC5A12 is expressed in the kidney, small intestine, and skeletal muscle and to a lesser level in brain and retina. SLC5A12 substrate specificity is similar to that of SLC5A8. However, the affinity of SLC5A12 for monocarboxylate substrates is lower than SLC5A8 (Srinivas et al., 2005).

Notably, the localization of these transporters is different at the tissue level suggesting different functions. In the kidney, SLC5A8 is expressed in the apical membrane of tubular epithelial cells in the S2–S3 proximal tubule segments where it is involved in renal reabsorption of lactate and pyruvate (Barac‐Nieto, Murer, & Kinne, 1980; Ganapathy et al., 2008; Gopal, Miyauchi, et al., 2007; Yanase, Takebe, Nio‐Kobayashi, Takahashi‐Iwanaga, & Iwanaga, 2008). Indeed, SLC5A8‐knockout mice exhibit increased urinary excretion of lactate (Frank et al., 2008). By contrast, renal SLC5A12 is highly expressed in the initial part of the proximal tubules and decreases toward the S3 segment. Thus, the proximal convoluted tubules provide low and high affinity transporters in the upper and lower proximal tubules, respectively (Gopal, Umapathy, et al., 2007).

In the brain, SLC5A8 mediates cellular uptake of lactate and ketone bodies by neurons (Martin et al., 2006), while SLC5A12 is specifically expressed by astrocytes. Besides the physiological functions, SLC5A8 has been reported as tumor‐suppressing molecule; its expression is in fact decreased in several tumors such as human colon cancer, papillary thyroid carcinomas, pancreatic cancer, prostate tumor, acute myeloid leukemia, and glioma formation (Ganapathy et al., 2008; Li et al., 2003; Park et al., 2008).

In the bowel, SLC5A8 is expressed in the lumen‐facing apical membrane of colonic and intestinal epithelial cells, while SLC5A12 is expressed primarily in the small intestinal tract (Gopal, Miyauchi, et al., 2007; Teramae et al., 2010).

The different tissue localization may indicate a distinct role of these transporters in regulating cell functions with important therapeutic implications.

## 3 LACTATE MODULATES IMMUNE CELL FUNCTIONS

It is becoming particularly attractive the concept that metabolites can act as immunomodulatory molecules regulating several immune cell functions (Figure 2; Haas et al., 2016). For instance, lactic acid has been reported to suppress the proliferation and cytokine production of human cytotoxic T lymphocytes (Fischer et al., 2007) via a mechanism that involves T cell receptor‐triggered phosphorylation of JNK, c‐Jun and p38, which is implicated in interferon‐γ (IFN‐γ) production (Mendler et al., 2012). In this context, our group has reported that lactic acid is able to modulate CD8^+^ but not CD4^+^ T cell responses (Haas et al., 2015; Pucino, Bombardieri, Pitzalis, & Mauro, 2017), whereas sodium lactate regulates CD4^+^ T cell migration and cytokine production without affecting CD8^+^ T cells.

It is well recognized that T cells display different metabolic requirements; with cytotoxic and effector T cells being more dependent on glycolysis for proliferation and cytokine production (Macintyre et al., 2014), while regulatory T cells (Tregs) rely more on oxidative phosphorylation (Gerriets et al., 2015; Michalek et al., 2011). Recently, Angelin et al. (2017) showed that the Treg transcription factor forkhead box P3 (FOXP3) is able to reprogram the metabolism of Tregs, allowing them to cope with low‐glucose and high‐lactate microenvironments, therefore escaping the anti‐tumor immune surveillance. This elegant study demonstrates that Tregs can withstand sustained exposure to elevated levels of lactate, whereas effector T cells are impaired. This is due to lactate‐mediated reduction of NAD^+^ availability that is crucial for effector T cells for their functions. Indeed cytotoxic and effector T cells reduce NAD^+^ to NADH via glyceraldehyde‐3‐phosphate dehydrogenase and require constant NAD^+^ recycling through LDH to sustain glycolysis. In conditions where extracellular lactate is abundant (i.e., during chronic inflammation) this metabolite is re‐converted to pyruvate (reversed LDH reaction) with generation of NADH and impairment of glycolytic flux (Leite et al., 2011; Pucino et al., 2019). Reduced glycolysis is particularly detrimental for effector and cytotoxic T cells, as it affects the ability to produce IFN‐γ (Chang et al., 2013) as well as their migratory capabilities (Haas et al., 2015; Pucino et al., 2019). On the other side, Tregs are less affected by reduced NAD^+^/glycolysis. In addition, FOXP3 is also able to regulate the direction of the LDH reaction in favor of the oxidation of lactate to pyruvate, leading to a decreased production of lactate by Tregs (as compared to conventional T cells) (Angelin et al., 2017). This is mechanistically related to FOXP3‐mediated suppression of the transcription factor Myc which in turn increases pyruvate dehydrogenase activity enhancing oxidative phosphorylation and NAD^+^ regeneration (Angelin et al., 2017).

LDH is required for CD4^+^ T cells to sustain aerobic glycolysis and for the production of IFN‐γ, enabling a proper differentiation to T helper 1 (Th1) cells (Peng et al., 2011). Genetic deletion of LDH isoform A (LDHA) in CD4^+^ T cells significantly reduced glucose consumption with a shift toward an oxidative metabolism, and decreased IFN‐γ expression. Moreover, the deletion of LDHA caused a reduction in the available pool of acetyl‐coenzyme A (acetyl‐CoA), which is important for histone acetylation. These data demonstrate that LDHA regulates INF‐γ production in Th1 cells, through an epigenetic mechanism of histone acetylation coupled with cellular metabolism (Peng et al., 2011).

Lactate has been shown to regulate immune responses during infections. Indeed, lactate serves as a key metabolite responsible for glycolysis‐mediated retinoic‐acid‐inducible gene I (RIG‐I)‐like receptors (RLRs), which is implicated in type 1 IFN production and viral clearance. Specifically, lactate induces an inhibitory signal by directly binding to mitochondrial antiviral‐signaling (MAVS) protein and preventing MAVS aggregation. Notably, lactate restoration reverses increased IFN production caused by lactate deficiency. LDHA inactivation and subsequent lactate reduction, using pharmacological and genetic approaches, increases type I IFN production and protects mice from viral infection (W. Zhang et al., 2019).

## 4 LACTATE: A KEY PLAYER IN CANCER

The tumor‐associated microenvironment consists of malignant cells, several immune cells, non‐cancer stromal cells, fibroblasts as well as increased angiogenesis and lymphangiogenesis (Galdiero, Varricchi, Loffredo, Mantovani, & Marone, 2018; Hanahan & Weinberg, 2011; Varricchi et al., 2018). A typical feature of the tumor microenvironment is its reduced pH to levels of ~6.5 (Corbet & Feron, 2017). Lactate is the main metabolite responsible for this acidosis as a consequence of high cellular glycolytic rate. Here lactate, transported through specific carriers expressed on the cell membrane of cancer and immune cells, and shuttling from cancer cells to the extracellular space, rewires intracellular metabolic pathways and suppresses immune responses enhancing tumor growth (Figure 2; Liu et al., 2019). Lactate is in fact an energy substrate for cancer cells in conditions of glucose deprivation. For instance, lactate is a carbon fuel molecule for the TCA cycle in some tumors such as the human non‐small‐cell lung cancers (NSCLCs), contributing to biomass synthesis for tumor growth (Faubert et al., 2017). Infusing human NSCLCs patients with ^13^C‐lactate has indeed revealed extensive labeling of TCA cycle metabolites. Deleting MCT1 from tumor cells suppressed lactate‐dependent metabolite labeling in mice. In addition, lactate contribution to the TCA cycle was predominant in comparison to glucose (Faubert et al., 2017). These data were further confirmed in the same year by Hui et al. who identified lactate as carbon fuel for TCA cycle, both in normal and cancerous tissues. This study showed that the infusion of ^13^C‐lactate, in fed and fasted mice, induced extensive labeling of TCA cycle metabolites in all tissues, and in lung and pancreatic tumors the contribution of circulating lactate to the activity of TCA cycle was greater than that of glucose. In addition, lactate promotes glutamate uptake and catabolism by increasing the expression of glutamine transporter ASCT2 and glutaminase 1 in cancer cells (Perez‐Escuredo, Dadhich, et al., 2016). These findings demonstrate that lactate is a main carbon supplier of the TCA cycle both in normal tissues and cancer, and that glycolysis and the TCA cycle are uncoupled at the level of lactate, allowing the independent regulation of the two processes (Hui et al., 2017).

The effects of lactate are not only due to its ability to feed metabolic pathways, but also to its ability to trigger a signaling pathway via its receptor G protein‐coupled receptor 81 (GPR81); a surface lactate receptor involved in the regulation of lipolysis (Lafontan & Langin, 2009) and in cancer cell survival (Roland et al., 2014). In this context, Feng et al. (2017) showed that lactate can regulate the expression of PD‐L1 in human lung cancer cells via GPR81. PD‐L1 is the ligand of PD1, a receptor expressed on the membrane of activated T cells, responsible for reduced proliferation and effector function of T cells, and a major target for cancer immunotherapy. The authors showed that lactate, via GPR81, upregulates the expression of PD‐L1 at a transcriptional level leading to suppression of the T cells effector functions in co‐culture experiments (Feng et al., 2017).

Lactate influences macrophage functions in the context of tumor environment. Tumor‐associated macrophages (TAMs) “sense” metabolic changes typical of the tumor microenvironment via the expression of MCTs. Cancer cells produce high amount of lactate, which is extruded in the intercellular space via MCT4. Released lactate is then taken up by tumor‐associated macrophages via MCT1, promoting in turn macrophage polarization toward a TAM (M2) phenotype with high expression of arginase 1 (Arg1), vascular endothelial growth factor (VEGF) production and subsequently tumor growth (Colegio et al., 2014; Figure 2). The authors found that lactate‐induced VEGF and Arg1 expression was dependent on HIF‐1α, as HIF‐1α‐deficient macrophages were not able to upregulate both VEGF and Arg1 upon lactate stimulation (Colegio et al., 2014). Notably, TNF‐α secretion by human monocytes was found to be suppressed in the presence of high lactate concentration and reduced pH in tumor microenvironment (Mendler et al., 2012). Neutralizing tumor acidity with bicarbonate monotherapy was able to impair the growth of some cancer types (Pilon‐Thomas et al., 2016). Similarly, another study demonstrated that lactate plays an important role in the communication between TAM and cancer cells undergoing epithelial‐to‐mesenchymal transition (Su et al., 2014).

Recently, it has been reported that lactate modulates the expression of macrophage‐specific vascular ATPase subunit ATP6V0d2, whose levels are regulated by transcription factor EB. The authors found increased mammalian target of rapamycin (mTOR) activation upon lactate stimulation suggesting that lactate could modulate ATP6V0d2 expression via this pathway (Liu et al., 2019). However, further investigation is needed to establish a key role of mTOR in lactate‐mediated ATP6V0d2 expression. Interestingly, Atp6v0d2−/− mice were susceptible to tumor growth, with enhanced HIF‐2α‐mediated VEGF production in macrophages that display a pro‐tumorigenic phenotype. These findings were also confirmed in humans. Indeed, in a cohort of patients with lung adenocarcinoma, expression of ATP6V0d2 and HIF‐2α was positively and negatively correlated with survival, respectively (Liu et al., 2019). The latter finding suggests a critical role of the macrophage lactate/ATP6V0d2/HIF‐2α axis in maintaining tumor growth in human patients. Furthermore, lactate can directly modulate angiogenesis allowing tumor growth. The lactate‐signaling pathway was reported to drive HIF‐1/VEGF activation independently of hypoxia in endothelial cells. MCT1 blockade was shown to have anti‐angiogenic effects together with reduced tumor growth in a model of colon cancer xenograft involving the co‐injection of human umbilical vein endothelial cells HUVECs (Vegran, Boidot, Michiels, Sonveaux, & Feron, 2011). Lactate has also an established role in the biology of cancer‐associated fibroblasts (CAFs) that are the major cellular stromal component of many solid tumors.

Ippolito et al. (2019) recently found that lactate uptake alters the NAD^+^/NADH ratio in the prostate cancer cells, which leads to increased mitochondrial mass and activity via an intracellular mechanism involving SIRT1‐dependent peroxisome proliferator‐activated receptor γ coactivator‐1 activation. The high exploitation of mitochondria results in tricarboxylic acid cycle deregulation, accumulation of oncometabolites and reactive oxygen species (ROS) generation. Additionally, using both in vitro and in vivo prostate cancer models the authors found that cancer cells hijack CAF‐derived functional mitochondria through the formation of cellular bridges.

In pancreatic cancer, lactate has been reported to promote posttranscriptional modifications that can contribute to tumor growth (Bhagat et al., 2019). Indeed, lactate produced by cancer cells increases the production of alpha‐ketoglutarate (aKG) within mesenchymal stem cells (MSCs). In turn, aKG‐mediated activation of the demethylase TET is responsible for decreased cytosine methylation and increased hydroxymethylation during de novo differentiation of MSCs to CAF. Notably, the co‐injection of neoplastic cells with TET‐deficient MSCs inhibited tumor growth in vivo proposing a new anti‐cancer therapeutic option (Bhagat et al., 2019).

### 4.1 MCTs: Expression in cancer

MCT1 expression has been reported in a variety of human malignancies including head and neck, lung, stomach, colon, prostate and cervix cancers, as well as gliomas (Afonso et al., 2015; Kennedy & Dewhirst, 2010; Miranda‐Goncalves et al., 2013; Pinheiro et al., 2012; Sonveaux et al., 2008). MCT1 has also been proposed to be the most important isoform responsible for lactate transport across the plasma membrane in breast and bladder cancer, NSCLCs and ovarian carcinomas (Afonso et al., 2015).

MCT4 is also widely distributed in different cancer types. Its expression has indeed been reported in breast, colon, bladder and prostate cancers, as well as in cancers of the gynecologic tract and gliomas (Afonso et al., 2015; Miranda‐Goncalves et al., 2013; Pinheiro et al., 2012).

Pertega‐Gomes and Baltazar (2014) reported a correlation between the expression of MCT1, MCT2 and MCT4, and the different stages of prostate cancer progression (Pertega‐Gomes & Baltazar, 2014). In another study focused at NSCLC, Eilertsen et al. (2014) described MCT1 as a prognostic and survival biomarker. In the same vein, the co‐expression of GLUT1 and MCT1 and of GLUT1 and MCT4 was found to be a negative prognostic factor associated with poor disease‐specific survival.

Besides MCTs, proton‐sensing GPRs such as T cell death‐associated gene 8 have also been shown to be important for the modulation of T cells in an acidic tumor environment and during inflammation suggesting that an interaction between MCTs and other sensing molecules is necessary to facilitate a proper shuttling activity (Ishii, Kihara, & Shimizu, 2005; Pilon‐Thomas et al., 2016). In line with this, it has been reported that intracellular carbonic anhydrase CAII can act as proton antennae facilitating proton‐driven lactate flux through MCTs in Xenopus oocytes. The knockdown of CAII with siRNA reduced MCT1‐mediated lactate transport in MCF‐7 breast cancer cells revealing a new potential therapeutic strategy (Noor et al., 2018).

## 5 LACTATE SUSTAINS CHRONIC INFLAMMATION IN IMMUNE‐MEDIATED INFLAMMATORY DISORDERS

While in tumor cells lactate plays a key role in suppressing T cell effector functions, in the inflammatory context lactate activates a “stop migration” signal in T cells leading to their accumulation at the inflammatory site (Figure 2). These events are mediated by sodium lactate and lactic acid uptake through the transporters SLC5A12 and SLC16A1, which are selectively expressed on the surface of CD4^+^ and CD8^+^ T cells, respectively (Haas et al., 2015). Interestingly, the inhibition of T cell migration is regulated via lactate interference with the glycolytic pathway (Droge, Roth, Altmann, & Mihm, 1987; Haas et al., 2015; Pucino et al., 2019). Indeed, CD4^+^ T cells, in the presence of sodium lactate, display a downregulation of several glycolytic enzymes and glucose flux (Haas et al., 2015; Pucino et al., 2019). Lactate can also modulate cytokine production. For instance, tumor derived lactate has been shown to enhance the IL‐23/IL‐17 pathway acting as a pro‐inflammatory signal (Shime et al., 2008; Yabu et al., 2011). Subsequently, it was found by our group that sodium lactate can directly modulate IL‐17 production by driving the plastic differentiation of CD4^+^ T cells in a Th17 subset in the inflamed tissue (Haas et al., 2015; Pucino et al., 2019). These findings have an important impact on the understanding of the role of lactate in the context of immune‐mediated inflammatory disorders (IMIDs, i.e., rheumatoid arthritis [RA]) where lactate may act as an inflammatory signal leading to the inhibition of CD4^+^ T cell migratory capability and to their differentiation in a Th17 subset thus sustaining the chronic inflammatory process (Figure 2). In addition, SLC5A12 has been found to correlate with the inflammatory score and T cell infiltration in RA synovial tissue, suggesting its possible role in the pathogenesis of this disease (Haas et al., 2015; Pucino et al., 2019).

Accumulation of lactate in the synovial fluid of RA patients is the result of the high metabolic demand of synovial cells (Garcia‐Carbonell et al., 2016). Even though there is not a clear correlation between lactate concentration in the synovial fluid and the disease activity, some authors found that synovial lactate level could be a reliable indicator of differentiating inflammatory arthritis (Gobelet & Gerster, 1984). Similarly, LDH isoenzymes were found to be higher in serum and synovial fluid of RA compared to osteoarthritis (OA) patients (Pejovic, Stankovic, & Mitrovic, 1992) and LDH activity was found to be increased in RA synovial tissues compared to healthy controls (Lindy, Uitto, Turto, Rokkanen, & Vainio, 1971).

The abundance of lactate in inflammatory sites, as result of the hypoxic environment, is responsible, at least in part, for the hypoxic‐induced signaling. Notably, lactate signaling and subsequent biologic responses appear to be functionally uncoupled from HIF‐1α‐induced metabolic reprogramming, by employing NDRG family member 3 (NDRG3) as a critical link (Lee et al., 2015). The authors found that the NDRG3 protein is degraded in a PHD2/VHL‐dependent manner in normoxia, similarly to HIF‐1α, but is protected from degradation by binding to lactate that accumulates under hypoxia. Indeed NDRG3 levels increase upon lactate stimulation irrespective of HIF‐1α. In addition, suppression of lactate production with either a LDHA pharmacologic inhibitor or LDHA silencing (siRNA) specifically inhibited the NDRG3 protein accumulation in a dose‐dependent manner. These events were reversed by the addition of lactate to the culture medium without affecting HIF‐1α protein levels. Moreover, NDRG3 promotes the switch toward a Th17 phenotype while it inhibits regulatory T cell differentiation (Shi et al., 2011). This might explain a possible mechanism through which lactate can modulate the balance between Th17 and Treg cells during hypoxia.

Opposite to T cells where it acts as a pro‐inflammatory molecule, on mast cells lactate has been reported to exhibit an anti‐inflammatory effect. Indeed, IL‐33‐mediated mast cell inflammatory cytokine production is inhibited upon lactate treatment (Abebayehu et al., 2016). These effects of lactate on mast cell activation were dependent on acidic pH and the MCT1 carrier. More recently, the same group reported that lactate inhibited lipopolysaccharide (LPS)‐induced cytokine and chemokine production by mast cells in vitro (Caslin et al., 2019). These events were linked to HIF‐1α‐dependent miR‐155 suppression. Indeed, HIF‐1α siRNA transfection in bone marrow‐derived mast cells prevented lactate‐mediated miR‐155‐5 suppression. Interestingly, lactate effects were mimicked by glycolytic inhibitors and reversed by increasing ATP availability (Abebayehu et al., 2016; Caslin et al., 2019).

The role that lactate may play in modulating macrophages in the context of IMIDs is controversial. It has been reported that LPS‐induced inflammatory responses in macrophages is reduced in the presence of lactate in a GPR81‐independent manner (Errea et al., 2016). While another study showed that lactate promoted macrophage activation upon TLR2 and TLR4 stimulations via MCT4, which regulates lactate export through the cell membrane. This was supported by observations that MCT4 knockdown attenuated the expression of pro‐inflammatory mediators in murine macrophages (Tan et al., 2015). This evidence indicates that lactate can modulate macrophage functions and fate engaging different signaling pathways. It would be interesting to investigate how these observations can differ in macrophages from healthy versus pathological conditions.

In this context, lactate‐derived lactylation of histone lysine residues has been recently identified as a new epigenetic modification that directly stimulates gene transcription from chromatin both in mouse and human. Using M1 macrophages that have been exposed to bacteria, the authors showed that histone lactylation has different temporal dynamics from acetylation. In the late phase of M1 macrophage polarization, increased histone lactylation induces homeostatic genes that are involved in wound healing, including Arg1. These findings highlight a homeostatic role for lactate during infections (D. Zhang et al., 2019).

Taken together these data support a role for lactate in the modulation of immune cell functions during inflammation. While the role of lactate in macrophage‐directed inflammation is still being debated, lactate behaves as a pro‐inflammatory molecule on T cells, leading to the production of cytokines necessary for the differentiation of specific T cell subsets and regulating their ability to migrate. Targeting lactate transporters, LDH, and HIF‐1α may represent a novel therapeutic intervention in IMIDs elicited by pro‐inflammatory T cells.

## 6 THERAPEUTIC STRATEGIES TARGETING LACTATE SIGNALING PATHWAY

Different approaches modulating lactate signaling pathway, such as MCTs and LDH inhibitors, have been demonstrated to be successful both in vitro and in vivo. The blockade of the lactate transporter MCT1 has shown to reduce the proliferation of breast cancer cells co‐expressing MCT1 and MCT4 (Hong et al., 2016), and decrease HIF‐1α‐induced angiogenesis in cervix squamous carcinoma (Sonveaux et al., 2008, 2012). In addition, MCTs inhibition with α‐cyano‐4‐hydroxycinnamic acid was reported to reduce lactate‐mediated inhibition of inflammatory gene expression and NF‐kappaB activity in human macrophages, indicating that lactate transport through monocarboxylate transporters is required for macrophage effector functions (Samuvel, Sundararaj, Nareika, Lopes‐Virella, & Huang, 2009).

In this context, AZD3965 (MCT‐1 inhibitor) has shown to be safe in a Phase 1 clinical study in the United Kingdom where it has been tested in solid tumor and B cell lymphoma patients (Curtis et al., 2017).

LDH has also been reported to be increased in tumor cells (Fantin, St‐Pierre, & Leder, 2006; Husain, Huang, Seth, & Sukhatme, 2013). Knocking down LDHA by short hairpin RNAs determined a reduced ability of tumor cells to proliferate under hypoxic conditions. This event was combined with increased mitochondrial respiration and decreased mitochondrial membrane potential. The tumorigenicity of the LDHA‐deficient cells was strongly reduced, and this phenotype was reversed by supplementation with the human LDHA (Fantin et al., 2006). LDHA‐depleted tumors display a decreased frequency of myeloid‐derived suppressor cells (MDSCs) and natural killer cells improved cytolytic function. The addition of exogenous lactate increased the frequency of MDSCs generated from murine bone marrow cells and inhibited cytolytic function of both human and murine NK cells in vitro, supporting the role of lactate in tumor immune surveillance escape (Husain et al., 2013). This evidence was also supported by Brand et al. (2016) showing that lactate leads to tumor immune escape by inhibiting the function and survival of T and NK cells. In the same context, another study has shown that tumor‐infiltrating liver‐resident NK cells display signs of mitochondrial stress, which are recapitulated in vitro by treating liver‐resident NK cells with lactate. Lactate‐mediated apoptosis and mitochondrial dysfunction in these cells was reverted by blocking mitochondrial ROS accumulation (Harmon et al., 2019).

Due to the emerging role of lactate in the field of inflammation and autoimmunity, lactate transporters are gaining much attention as novel therapeutic targets even in these contexts. In this regard, it has been found that MCT4 is upregulated by RA synovial fibroblast (RASFs) compared to OA SF (Fujii et al., 2015). Silencing of MCT4‐inhibited proliferation of RASFs in vitro and reduced the severity of arthritis in a mouse model of collagen‐induced arthritis in vivo (Fujii et al., 2015). Similarly, MCT4 is required for macrophage activation upon TLR2 and TLR4 stimulations as confirmed by reduced expression of pro‐inflammatory molecules, such as TNF‐α and IL‐6, in MCT4 knockdown macrophages. In addition, these events were accompanied by intracellular accumulation of lactate and decreased glycolysis (Tan et al., 2015).

In addition, the blockade of SLC5A12 (with shRNA or antibody) restored T cell functions in vitro as well as ameliorated disease severity in vivo in mouse models of peritonitis and arthritis (Haas et al., 2015; Pucino et al., 2019) suggesting a novel therapeutic target to reduce inflammation in IMIDs.

## 7 CONCLUDING REMARKS

The mechanisms regulating the crosstalk between metabolism and the immune system have been investigated in some depth in the last few years (Buck, Sowell, Kaech, & Pearce, 2017; Geltink, Kyle, & Pearce, 2018). In this context, lactate has emerged as an important signaling and immunomodulatory molecule, able to promote inflammatory responses and suppress anti‐tumor response. The discovery of lactate transporters on the surface of immune cells has opened novel and important perspectives for the understanding of the pathogenesis of inflammatory disorders and tumors. Targeting these molecules might prove crucial in the modulation of immune cell functions to achieve beneficial therapeutic effects.

## CONFLICT OF INTEREST

The authors have declared no conflicts of interest for this article.

## AUTHOR CONTRIBUTIONS

**Michelangelo Certo**: Conceptualization; data curation; formal analysis; funding acquisition; investigation; methodology; project administration; resources; software; supervision; validation; visualization; writing‐original draft, review and editing. **Giancarlo Marone**: Conceptualization; data curation; formal analysis; funding acquisition; investigation; methodology; project administration; resources; software; supervision; validation; visualization; writing‐original draft, review and editing. **Amato de Paulis**: Conceptualization; data curation; formal analysis; funding acquisition; investigation; methodology; project administration; software; supervision; validation; visualization; writing‐original draft, review and editing. **Claudio Mauro**: Conceptualization; data curation; formal analysis; funding acquisition; investigation; methodology; project administration; resources; software; supervision; validation; visualization; writing‐original draft, review and editing. **Valentina Pucino**: Conceptualization; data curation; formal analysis; funding acquisition; investigation; methodology; project administration; resources; software; supervision; validation; visualization; writing‐original draft, review and editing.

## RELATED WIREs ARTICLE

https://dx.doi.org/10.1002/wsbm.1397
